# DPOAEs and tympanal membrane vibrations reveal adaptations of the sexually dimorphic ear of the concave-eared torrent frog, *Odorrana tormota*

**DOI:** 10.1007/s00359-022-01569-8

**Published:** 2022-09-15

**Authors:** Ariadna Cobo-Cuan, Albert S. Feng, Fang Zhang, Peter M. Narins

**Affiliations:** 1 Department of Integrative Biology and Physiology, University of California Los Angeles, Los Angeles, CA 90095 USA; 2 Department of Molecular and Integrative Physiology and Beckman Institute, University of Illinois at Urbana-Champaign, Urbana, IL 61801 USA; 3 College of Life Sciences, Anhui Normal University, Wuhu, Anhui China; 4 Department of Ecology and Evolutionary Biology, University of California Los Angeles, Los Angeles, CA 90095 USA

**Keywords:** Hearing, Amphibian, Tympanum vibration, DPOAE, *Odorrana tormota*

## Abstract

While most anuran species are highly vocal, few of them seem to be endowed with a complex call repertoire. *Odorrana tormota*, combines a remarkable vocalization complexity with auditory sensitivity over an extended spectral range spanning from audible to ultrasonic frequencies. This species is also exceptional for its ability to modify its middle ear tuning by closing the Eustachian tubes (ET). Using scanning laser Doppler vibrometry, the tympanal vibrations were measured to investigate if the tuning shift caused by the ET closure contributes to intraspecific acoustic communication. To gain insight into the inner ear frequency selectivity and sensitivity of this species, distortion product otoacoustic emissions were recorded at multiple frequency-level combinations. Our measurements of inner ear responses indicated that in *O. tormota* each sex is more sensitive to the frequencies of the other sex's vocalizations, female ears are more sensitive to 2–7 kHz, while male ears are more sensitive to 3–15 kHz. We also found that in both sexes the ET closure impacts the sensitivity of the middle and inner ear at frequencies used for communication with conspecifics. This study broadens our understanding of peripheral auditory mechanisms contributing to intraspecific acoustic communication in anurans.

## Introduction

It was in 2000 that two of us (ASF and PMN) first visited Huangshan Hot Springs, China to record the vocalizations of the Concave-eared torrent frog, *Amolops tormotus*, aka *Odorrana tormota*. Much has been learned in the nearly quarter-century since then about the auditory system and vocal behavior of this remarkable species and its congenerics. Unlike most of the frogs that have a small repertoire of stereotyped calls, *O. tormota* produces an extremely large repertoire of calls (Feng et al. 2002, 2009; Narins et al. 2004; Zhang et al. 2017). This is one of the few anuran species where both males and females produce mating vocalizations (Given 1987; Emerson 1992; Tobias et al. 1998; Shen et al. 2008). Their calls are sexually dimorphic and often exhibit many ultrasonic harmonics and distinct nonlinear phenomena (NLP) including extremely rapid frequency jumps, deterministic chaos, and multiple subharmonics (Suthers et al. 2006; Feng et al. 2009; Zhang et al. 2017). In addition, and most unusually, the males are capable of behaviorally responding to the ultrasonic components of conspecifics’ vocalizations (Narins et al. 2004; Feng et al. 2006; Feng and Narins 2008).

**Fig. 1:**
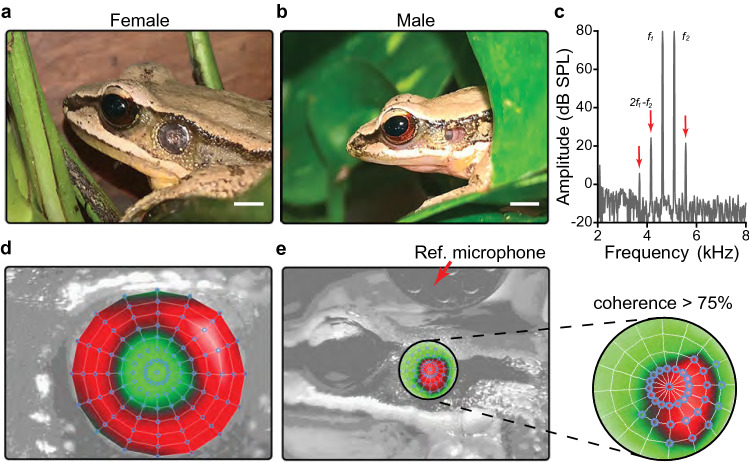
Photographs of adult female (**a**) and male (**b**) of *Odorrana tormota* illustrate the sexual dimorphism of the middle ear. In males, the tympanum is inside a cavity and only its ventrocaudal portion is visible through the ear canal aperture. Scale bars represent 3 mm. **c** Spectrum of the microphone signal showing the stimuli (*f*_1_ and *f*_2_) and the DPOAE spectral peaks (indicated by red arrows). Distortion product DP2*f*_1_*–f*_2_ is labelled by its frequency. *L*_1_ = *L*_2_ = 80 dB SPL, *f*_2_/*f*_1_ = 1.1, Examples of radial grids of scanning points used to evaluate the tympanum response in females (**d**) and males (**e**). Only measures with > 75% coherence between the vibrometer and microphone signals were processed. In the deflection shapes, the red color represents movement of the tympanum toward the reader and the green color represents movement of the tympanum away from the reader

The Concave-eared torrent frogs have a distinctive sexual dimorphism in the auditory periphery (Fig. 1a, b). Contrasting nearly all other frogs, males of *O. tormota* possess ear canals, their tympanic membranes are recessed and thus closer to the inner ear (Fig. 1b). These morphological adaptations may play a role in facilitating the transmission of high frequencies through the middle ear (Feng et al. 2006). Unlike males, females do not have recessed ears. Males are also remarkable because they can dynamically modify the auditory sensitivity of their middle ear by controlling the closing of its normally open Eustachian tubes (ET) (Gridi-Papp et al. 2008). With the discovery of this fascinating mechanism new questions arose. Is this dynamic auditory tuning exclusive to males? Does the tuning shift in the middle ear of *O. tormota* contribute to the intraspecific acoustic communication? Is the inner ear sensitive to those changes in frequency sensitivity of the tympanum?

In this study, we examined the effect of the open/closed Eustachian tubes on the peripheral auditory tuning of both sexes of *O. tormota*. The vibrational behavior of the sexually dimorphic tympanal membrane was evaluated using scanning laser Doppler vibrometry. Tympanal vibrations elicited by both synthetic sounds and playback of intraspecific calls were assessed. We evaluated the frequency selectivity and sensitivity of the inner ear by recording Distortion Product Otoacoustic Emissions (DPOAEs).

## Material and methods

### Subjects

Adult *O. tormota* (Fig. 1a, b) were collected along the banks of the Fu Creek in Huangshan, China (118° 8′ 44.89′′ E, 30° 5′ 1.61′′ N; Elevation: 600 m) between 3 and 17 April 2019. DPOAE experiments were performed in 20 animals (10 non-gravid females and 10 males) in a quiet room near the collection site. Eight additional animals (four females and four males) were transported to the University of California Los Angeles (UCLA) where the vibrometric measurements were obtained from the tympanic membranes. In the UCLA lab, frogs were same-sex group-housed in polycarbonate aquaria under a 12-h light/dark cycle at 20–22 °C and fed live crickets twice weekly. Both males and females emitted typical calls, indicating a good general condition. Mean body weight was 16.72 ± 2.73 g in females and 3.19 ± 0.37 g in males; and mean snout-vent length was 57.93 ± 1.96 mm in females and 33.69 ± 1.54 mm in males. Housing and experimental procedures complied with IACUC standards and were approved by the UCLA Animal Care and Use Committee (protocol no. 1994-086-71).

### Vibrometric recordings

Tympanal vibrations were studied following the same general procedure used in a previous study on *O. tormota* (Gridi-Papp et al. 2008). Experiments were carried out on a vibration isolation table located in a dedicated acoustic isolation booth (Industrial Acoustics IAC series 1204A). A scanning laser Doppler vibrometer (Polytec PSV-300) with an OFV-056 scanning head was used to evaluate the tympanic membrane (TM) motion in response to ipsilateral acoustic stimulation. Each frog was orientated such that the TM was in focus and perpendicular to the laser beam. Acoustic stimuli were broadcast with a multi-field loudspeaker (MF1, Tucker-Davis Technologies) positioned at 6 cm from the frog tympanum and at 53° to the frog body axis. A reference microphone (MK 301, Microtech Gefell GmbH) connected to a conditioning amplifier (Brüel & Kjær Nexus 2690) was placed with its diaphragm parallel to the tympanum and near (ca. 5 mm) the tympanic annulus. The vibration velocity of the TM and sound level pressure were measured simultaneously and digitized through a data acquisition board (National Instruments, PCI-6110).

Vibrations were measured along a grid of scanning points with a radial distribution (Fig. 1d, e). To estimate the data reliability, the magnitude-squared coherence between the vibrometer and microphone signals was computed for each data point using a standard transfer function formula (Windmill et al. 2005). Only highly coherent measurements (> 75%) with minimal contamination by unrelated noise were averaged to calculate the vibration velocity spectrum that was used in the post-hoc analysis. This procedure allowed validating the scanning points that were over the tympanum and was particularly relevant in males, where only the ventrocaudal portion of the tympanum is visible through the ear canal aperture when it is positioned at an angle normal to the laser incidence (Fig. 1b, e).

To determine if in *Odorrana* females the auditory sensitivity of the middle ear changes with the ET closure as in males, we use a protocol of acoustic stimulation with pure tones similar to the one described by Gridi-Papp et al. (2008). Stimuli consisted of tone bursts (20-ms duration, 5-ms rise/fall times, 10-ms interval) spanning 1–40 kHz with a 1 kHz step size. Tones were calibrated to reach the position of the frogs’ TM at sound levels of 70 dB SPL.

To better characterize the male tympanum response to behaviorally relevant stimuli, representative calls of *O. tormota*, with and without nonlinear phenomena (NLP) (Feng et al. 2009), were broadcast in free-field acoustic conditions. Two male call-types (short call with NLP and two-note call) and one female call-type (long call with NLP) were selected. The multi-harmonic call without NLP was also presented in a modified version with equalized harmonics. The energy of the second and third harmonic was adjusted to equal the energy of the first harmonic using the graphic synthesizer of Avisoft-SASLab Pro software (Avisoft Bioacoustics). The intensity of the acoustic stimuli was 65–70 dB SPL at the frog position. Stimulus generation and data acquisition were controlled by Polytec Scanning Vibrometer software (version 8.3, Polytec Gmbh). Subsequent analysis was performed using custom software written in Matlab R2016b (The MathWorks, Inc.).

### DPOAE recordings

DPOAE measurements were collected within the first two days after frogs’ capture (Fig. 1c). Animals were anaesthetized with an intramuscular injection of a pentobarbital sodium solution (Nembutal, Ovation Pharmaceuticals, Inc., 50 mg/ml: ~ 1–1.2 µl/g body weight) in one of the hind limbs. Auditory measurements began approximately 30–45 min after Nembutal injection when the individuals reached areflexia (absence of toe-pinch or eye reflex). Anesthesia typically lasted for the entire course of the experiment, and thus no maintenance dose was necessary. During the acoustic evaluation, frogs were positioned in their natural sitting posture, and covered with wet gauze to prevent desiccation and to facilitate cutaneous respiration. Room temperature ranged from 14 to 18 °C. After the end of the experiment, the frog was removed from the setup and brought to a recovery container where it could easily be monitored. The next day, after the frog had fully recovered, it was released at its original collection site.

Acoustic signals were delivered to and recorded from the right ears exclusively. A custom-built, 3D-printed probe was carefully sealed over the skin surrounding the tympanic membrane. The probe included a port for a microphone (MK 301, Microtech Gefell GmbH: − 46 dB re 1 V/Pa, 5 Hz to 100 kHz) to record the DPOAE responses. Two multi-field loudspeakers (MF1, Tucker-Davis Technologies) were also coupled to the probe through 1-cm pieces of tygon tubing.

Stimuli were generated and responses were acquired using an RME Fireface UC audio interface (128 kHz, 24 bit). An amplifier (MN920, Microtech Gefell GmbH) provided 32 dB of gain for the microphone signal. Stimulus generation, data collection and analyses were performed using custom software written in Matlab R2016b (The MathWorks, Inc.). To ensure defined stimulus levels, the speakers were separately calibrated in situ using band-pass filtered (0.2–60 kHz) white noise. Sound pressure levels are given in dB SPL relative to 20 µPa.

DPOAEs were evoked with two simultaneous, in-phase pure tones of 4.16-s duration (1-ms rise/fall time) with an inter-stimulus interval of 500 ms. The frequencies and levels of these (primary) tones are denoted as *f*_1_, *f*_2_, and *L*_1_, *L*_2_, respectively. The amplitude of the distortion product at the frequency 2*f*_1_–*f*_2_ (termed DP2*f*_1_–*f*_2_) was obtained by performing an FFT on a time-domain-averaged (*n* = 65) signal using 8192 points (Fig. 1c). The background noise level was calculated as the mean amplitude of 5 FFT bins on either side of and 30 Hz from the 2*f*_1_–*f*_2_ frequency. To qualify as a valid DPOAE recording, the DP2*f*_1_–*f*_2_ amplitude at each frequency had to be at least two standard deviations above the background noise (≥ noise + 2SD).

DP-grams (DP2*f*_1_–*f*_2_ amplitude as a function of *f*_1_ frequency) were recorded with *f*_2_ frequencies from 1 to 18 kHz (in 200-Hz steps) and stimulus levels equal to 80 dB SPL. A fixed *f*_2_/*f*_1_ ratio of 1.1 and equal primary tone levels (*L*_1_ = *L*_2_) were used since these stimulus parameters have been shown to be optimal to evoke large DPOAE levels in frogs (van Dijk et al. 2002; Meenderink and Van Dijk 2006). To evaluate the DPOAE threshold curve, a matrix of frequency-level tone combinations was presented pseudo-randomly with *f*_2_ values between 1 and 9 kHz in females and between 2 and 18 kHz in males (in 1-kHz steps), and primary tone level values between 50 and 90 dB SPL (in 2-dB steps). The DP2*f*_1_*–f*_2_ amplitude for each stimulus frequency-level combination was represented in a heat map. The DPOAE threshold curve was delimited by the interpolated stimulus levels necessary to elicit a DP2*f*_1_*–f*_2_ amplitude ≥ noise + 2SD.

### Manipulations of the Eustachian tube

**Fig. 2:**
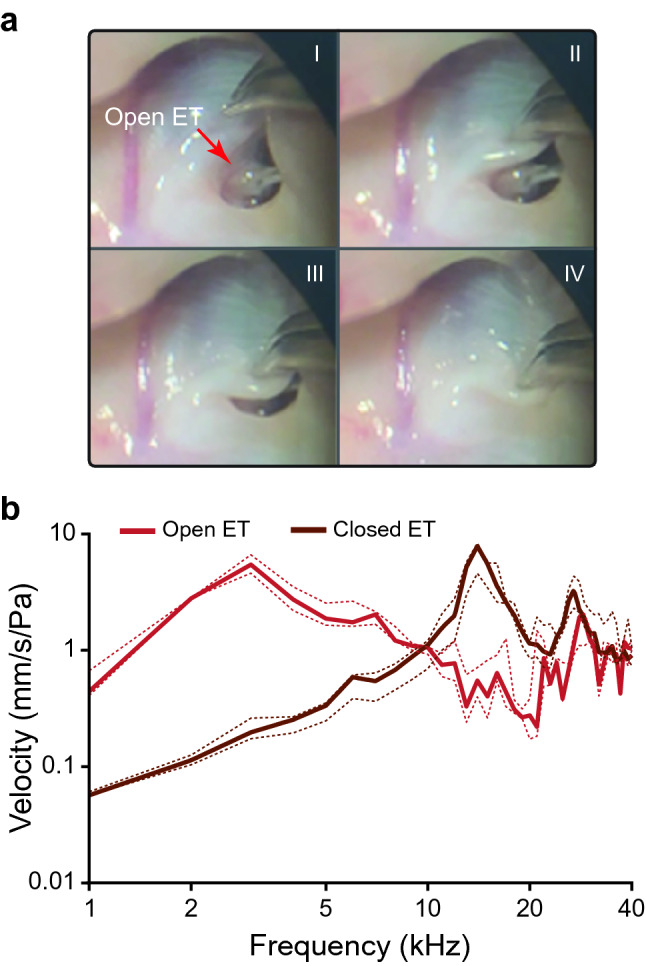
**a** Serial photographs of the procedure to close the eustachian tube (ET) in a female of *Odorrana tormota*. The tissue overlying the hyoid cartilage of the ET is gently grasped with forceps and moved toward the lumen until it is completely closed before applying Vetbond (not shown). **b** Velocity amplitude of the tympanic membrane of females with the ET open (red) and closed (brown). Stimuli were pure tones of 1–40 kHz with a 1 kHz step size. Solid lines denote the median values and dotted lines indicate the 25th and 75th percentiles

Most DPOAE and vibrometric measurements in the lab were obtained with the Eustachian Tube (ET) open, corresponding to the ET state most commonly observed in the field (Gridi-Papp et al. 2008). In some individuals, measurements were repeated with the ET closed to evaluate the effect of ET closure on the tuning of the tympanic membrane and on the DPOAE response. To achieve the ET-closed condition, the hyoid cartilage was extended over the lumen of the ET and glued (Vetbond) to the buccal skin covering the rostro-lateral border of the tube (Fig. 2a).

## Results and discussion

### Changes in tympanum sensitivity in *Odorrana* females

In females of *O. tormota*, we found that the auditory sensitivity of the middle ear changes with the ET closure as in males. The morphology that allows closing the ET is also similar to that described in males (Gridi-Papp et al. 2008). The distal end of the hyoid horn forms a hinge at its attachment to the skull that allows it to pivot. This pivoting mechanism allows the cartilaginous horn of the hyoid to arch over the lumen of the ET and close it. The capability of females to close the ET was discovered using anatomical observations and manipulations, much like was done for males of this species (Gridi-Papp et al. 2008). In subsequent behavioral observations of captive female frogs, we did not notice closure of the ET during vocalization, and only once only during swallowing. Further investigation is needed to determine whether female *Odorrana* frogs use the same neuromuscular mechanism as males to close the ET.

The tympanum velocity in response to acoustic stimulation with pure tones was evaluated in four females with the ET open and closed. With the ET open, the tympanum responded with vibration velocities above 1 mm/s/Pa over a broad range of frequencies (2–10 kHz); maximum velocities occurred at 3 kHz (Fig. 2b, red curve). With the ET closed, the tympanum response exhibited a shift in sensitivity towards a higher spectral range (10–21 kHz) and peak velocities occurred at 14 kHz (Fig. 2b, brown curve). This shift in middle ear tuning was described previously in males: a high sensitivity at frequencies < 10 kHz with the ET open changed to 10–32 kHz after the closure of the ET (Gridi-Papp et al. 2008). In both sexes, the ET closure tends to attenuate low frequencies and amplify higher frequencies, but the effects this mechanism could have in sound perception could differ widely considering the sexual differences in the anatomy and physiology of the auditory system of this species (Shen et al. 2008).

### Middle ear sensitivity to natural stimuli

**Fig. 3:**
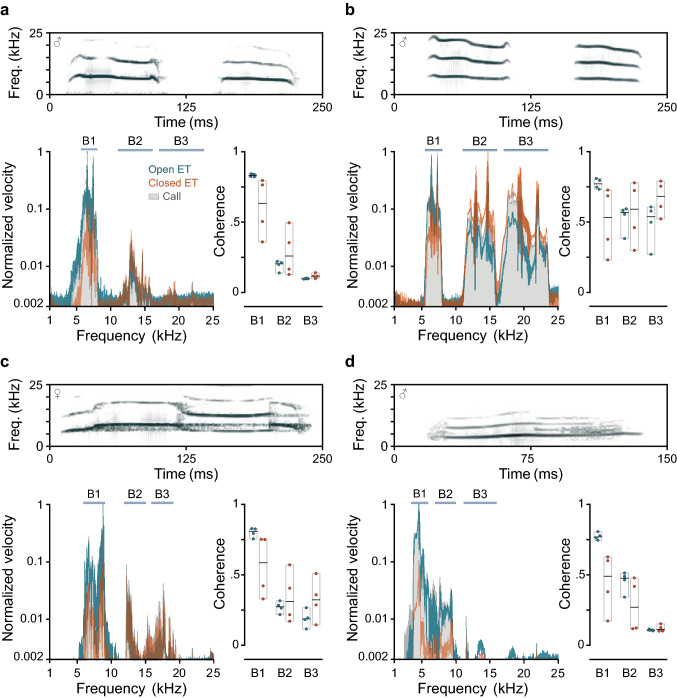
Tympanum response of males of *Odorrana tormota* to species-specific calls. Spectrograms of the vocal signals used as stimuli and velocity profiles of the tympanic membrane in response to them: **a** multi-note short-call (male), **b** multi-note short-call with equalized harmonics (male), **c** long-duration call (female), **d** short-duration call (male). Dark green: open-ET condition, orange: closed-ET condition. Normalized power spectra of the calls as recorded by the reference microphone are shown as gray areas. Scatter plots represent mean coherence between the stimulus (natural or synthetic call) and the tympanum vibration for the selected spectral bands (B1–B3, gray bands on top of spectra). Bold horizontal lines denote the median, and the bottom and top edges of the box indicate minimum and maximum, respectively

The tympanum’s sensitivity to conspecific calls was investigated in four males with the ET open and closed (Fig. 3). To assess the frequency-dependent relative differences in sensitivity between these conditions, we selected three spectral bands (B1–B3). For the multi-harmonic calls (natural and equalized), the analysis focused on the spectral bands centered around the dominant frequency (5.5–8 kHz) and around the two first harmonics (11–16 kHz, 17–23.5 kHz). For calls with NLP, we defined analysis bands around the three main spectral peaks which appeared at 6–9, 12–15 and 16–18 kHz in the female call, and at 3.5–6, 7–10 and 11.2–16 kHz in the male call.

In general, the frequency of the maximal vibrational response corresponded to the dominant frequency of the calls indicating that males’ tympana are mechanically well-tuned to the conspecific calls. The mean coherence between the vibration velocity of the tympanum and the sound pressure recorded by the reference microphone adjacent to the tympanum was also calculated at the specified spectral bands (Fig. 3). The highest coherence corresponded to the low-frequency band (B1) both in the ET-open state and in the ET-closed state. However, B1 coherence values corresponding to the recordings with the ET open (~ 0.8) were always higher relative to the recordings with the ET closed, where greater variability was also found (0.4–0.6). In contrast, median coherence values for the other spectral bands (B2 and B3) tend to be higher in the recordings with the ET closed. It is remarkable, however, that the changes in sensitivity caused by the closure of the ET, loss of sensitivity at low frequencies and gain of sensitivity at high frequencies, are not equivalent, and the relationship between them varies with the type of calls.

For example, when we analyzed the tympanum response to a multi-harmonic call for which the harmonic amplitudes roll off as the frequency increases, we found that the decline in the response to the fundamental frequency is not counteracted by an increase in the detection of the third harmonic, for which there was almost no vibration with either the open or closed ET (Fig. 3a). The use of equal-amplitude tones in experimental approaches helps to assess the spectral sensitivity of the auditory structures, but still falls short of illustrating their performance in a natural context. As with tones of equal amplitude (Gridi-Papp et al. 2008), the tympanum response to equalized harmonics suggests that the ET closure facilitates the detection of higher harmonics (Fig. 3b). However, the energy content of these high-frequency harmonics in the natural calls could be so low that they do not cause the eardrum to vibrate significantly (Fig. 3a). The capacity of detecting the higher harmonics potentially benefits from the ET closure but it would depend on the energetic roll-off characteristic of the call.

Our assessment of the tympanum’s vibration also used representative calls with NLP from both sexes. In the ET-closed condition, the response of the tympanum to the female call increased for frequencies greater than 10 kHz relative to the response corresponding to ET-open (Fig. 3c). In contrast, the tympanum response to the male call, which concentrates most of the energy below 10 kHz, mostly diminished and did not benefit from the ET closure (Fig. 3d). Generally, dominant frequencies of female calls are in a higher spectral range relative to male calls (~ 7.5–9.2 kHz, Shen et al. 2008; Zhang et al. 2017); consequently, harmonics and NLP appear at very high frequencies (> 15 kHz). These findings suggest that the closure of the ET in males of *O. tormota* could be relevant in a behavioral context beyond helping to extract the desired signals from background noise. It might play a role in intraspecific acoustic communication by helping to better detect the high-frequency components of female calls while decreasing sensitivity to low-frequency calls mainly produced by other males.

### DPOAEs in *O. tormota*

**Fig. 4:**
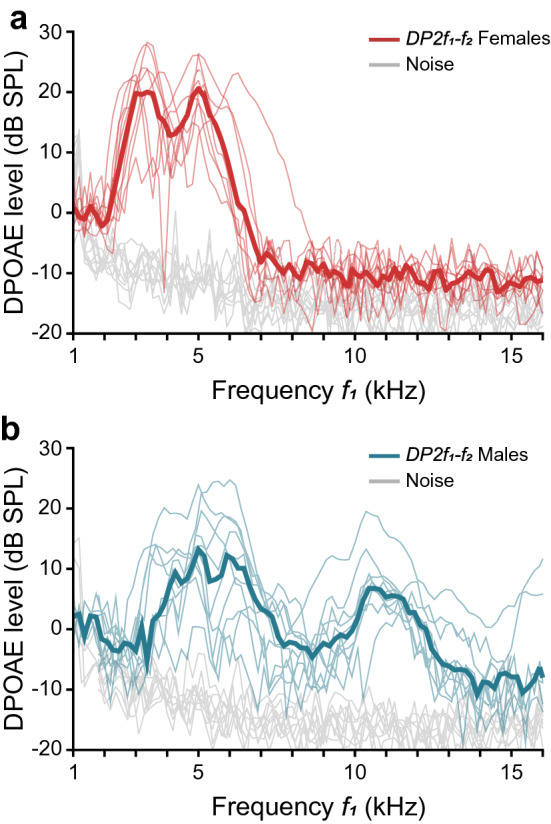
DPOAE response in *Odorrana tormota*. Individual DP-grams recorded in 10 females (**a**) and 10 males (**b**) reveal the sexual differences in the inner ear sensitivity. Data recorded with the ET open. Bold lines represent median DP-grams. System noise is indicated in gray. *L*_1_ = *L*_2_ = 80 dB SPL; *f*_2_/*f*_1_ = 1.1

Distortion product otoacoustic emissions were recorded in all 20 ears investigated (10 females, 10 males). A median DP-gram for each sex was obtained by determining the median DP2*f*_1_–*f*_2_ amplitude for each *f*_1_ frequency across same-sex subjects (Fig. 4). In females, the DP2*f*_1_–*f*_2_ was distinguishable from system noise (≥ noise + 2SD) when *f*_1_ was between 2.27 and 6.73 kHz, except for one individual in which the DPOAE response extended to 8.36 kHz. In males, DP2*f*_1_–*f*_2_ was observed in a more extended frequency range, from 3.09 to 14.73 kHz. In two males, DPOAE response was detected as high as 18.19 kHz, the highest *f*_1_ frequency used.

In most of our DPOAE analyses, we refer to the primary frequency *f*_1_, which is commonly used in the literature, thus allowing direct comparison with other species. Nevertheless, also revealing the frequencies at which the cubic distortions 2*f*_1_–*f*_2_ and 2*f*_2_–*f*_1_ were detected helps to grasp the remarkable extent of the hearing range in *O. tormota*. The DPOAE response in females appeared from 2 to 7.4 kHz, while in males this spectral range extended between 2.5 and 20 kHz. The inner ear of this species exhibits a reciprocal matched filtering (Cobo-Cuan and Narins 2020) that could facilitate the detection of the opposite sex's vocalizations. Sexual differences found in inner ear tuning are in agreement with the data from behavioral, vibrometric and electrophysiological evaluations of *O. tormota* (Gridi-Papp et al. 2008; Shen et al. 2011).

The bimodal DP-grams typically recorded in frogs (Van Dijk and Manley 2001; Van Dijk et al. 2003; Meenderink and Van Dijk 2004) are also confirmed in the DP-grams of *O. tormota* females. They exhibit two frequency regions with elevated DPOAE amplitudes which are separated by a notch in DPOAE amplitude. The two relative amplitude maxima reflect two spectral filters as derived from the amphibian papilla (AP) and the basilar papilla (BP). The best sensitivity in the lower frequency range, presumably corresponding to the AP, was found at 3.36 kHz (2.91–3.81 kHz). The second sensitivity peak, presumably corresponding to the BP frequency range, showed the best sensitivity at 5 kHz (4.73–6.19 kHz).

In males, however, it is noteworthy that most DP-grams exhibited three sensitivity peaks instead of two. The best sensitivity was found at 5.05 kHz (3.64–7.55 kHz) and at 7.64 kHz (6.36–10.45 kHz) in the low- and mid-frequency regions, respectively. The sensitivity peak in the high-frequency region appeared at 11.27 kHz (10.55–16.09 kHz). The presence of three relative amplitude maxima at low-, mid- and high-frequency regions was also described in DPOAE recordings from males of *Huia cavitympanum* (Cobo-Cuan et al. 2020). It is tempting to assume that the three spectral ranges of sensitivity may be related to three separate functional epithelial patches in the inner ear. To this end, it is important to consider that the AP itself appears to be a combination of two distinctly functioning auditory epithelia that fuse during development to form a single organ (Li and Lewis 1974). These AP regions have oppositely polarized hair-cell populations that differ in their electrical properties and ionic currents (Smotherman and Narins 1999). Interestingly, the AP caudal patch exhibits an elongation whose shape, relative length, and proportion of hair cells apparently correspond to the extension of auditory sensitivity to higher frequencies (Lewis et al. 1992). It is possible that in *O. tormota*, like in *H. cavitympanum*, DPOAEs corresponding to the low- and mid-frequency ranges are generated in the rostral and caudal regions of the AP, respectively. Compared to most anurans, these two species are exceptional in their ability to communicate over an extensive spectral range that reaches high frequencies. It could be possible that frogs’ species with auditory sensitivity over a wider spectrum have less overlapping between the frequency ranges detected by the two regions of the amphibian papilla. Further studies are necessary to test this hypothesis. Both the AP and the BP were tuned to significantly higher frequencies in males than in females (AP: *U* = 3, *p* < 0.001; BP: *U* = 0, *p* < 0.001).

**Fig. 5:**
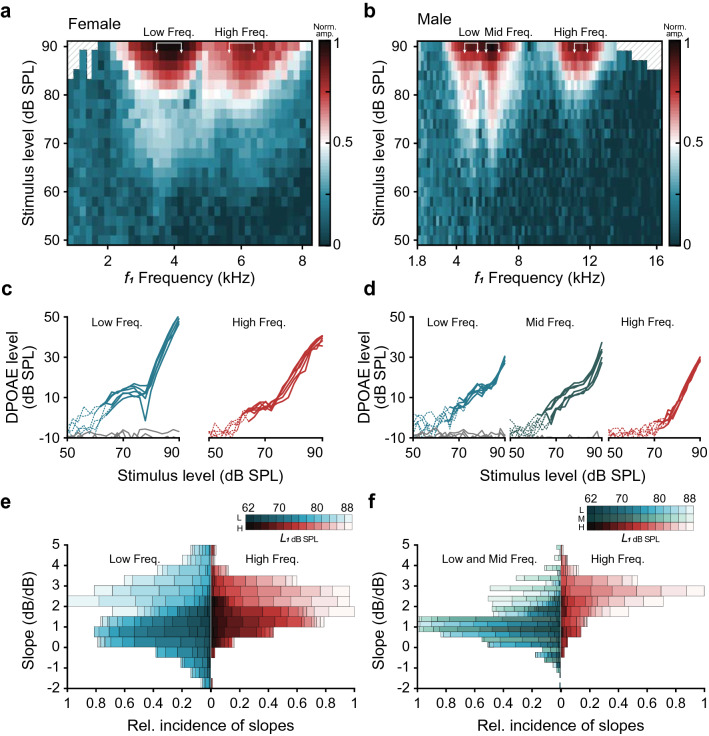
Frequency tuning and sensitivity of the inner ear. DPOAE responses recorded in a female (**a**) and a male (**b**) of *Odorrana tormota*. Normalized colormaps show the DP2*f*_1_*-f*_2_ amplitude for each frequency-level combination. The white arrows delimit the frequencies selected to evaluate DPOAE growth. Regions with diagonal-lines pattern indicate stimuli combinations that generated system distortions **c**, **d** DPOAE growth functions extracted from each sensitivity peak found in the corresponding DPOAE response matrices shown above. Solid lines correspond to DP2*f*_1_*–f*_2_ amplitude ≥ noise + 2SD. Background noise is indicated in gray. In both sexes, the growth functions were analyzed for low- and high-frequency regions, but mid-frequency regions were additionally evaluated in males. **e**, **f** Histograms including all the slopes of DPOAE growth registered at the different frequency ranges of high sensitivity in all individuals. Slopes’ distributions corresponding to low- and mid-frequency regions appear to the left, and slopes’ distributions corresponding to high-frequency regions appear to the right. DPOAE amplitude grows steeply (> 2 dB/dB) with high stimulus levels (> 80 dB SPL, lighter colors) while it decreases or grows with shallow slopes (≤ 1 dB/dB) with low stimulus levels (< 75 dB SPL, darker colors)

Examples of matrices of the DP2*f*_1_–*f*_2_ amplitude in response to a set of primary-tone frequency-level combinations are shown as color maps in Fig. 5a, b. From these response matrices, DPOAE threshold tuning curves were obtained by interpolating the stimulus levels necessary to elicit a DP2*f*_1_*–f*_2_ amplitude ≥ noise + 2SD across the frequency range tested. A Wilcoxon signed-rank test indicated that the minimum thresholds corresponding to each auditory papillae were not statistically different in females. The median minimum threshold of both the AP and the BP was 62 dB SPL. To examine differences in sensitivity between the auditory end-organs of males, only the minimum thresholds in the low- and high-frequency regions were compared. A Wilcoxon signed-rank test indicated that the AP minimum thresholds in males were significantly lower than the BP minimum thresholds (*Z* = 2.203, *p* = 0.027). The median of the minimum threshold was 63 dB SPL for the AP (60–64 dB SPL) and 66 dB SPL for the BP (62–72 dB SPL). These differences in sensitivity between papillae have been found in other anurans such as *Rana pipiens* and *H. cavitympanum*) (Meenderink and Van Dijk 2004; Cobo-Cuan et al. 2020). The involvement of active processes in the DPOAE generation could explain the higher sensitivity of the AP (Meenderink and Van Dijk 2006).

DPOAE growth functions (DPOAE amplitude as a function of the stimulus level) were extracted from the individual DPOAE response matrices at ± 0.5 kHz around the frequencies of highest sensitivity (Fig. 5c, d). The slope of the growth functions at each data point was calculated by fitting a straight line through its two neighbors (i.e., the data points evoked with stimulus levels 2 dB above and below the data point in question). Frequency distributions of the slopes corresponding to each sensitivity peak are depicted as histograms in Fig. 5e, f. In females, the growth functions were evaluated for low- and high-frequency regions, and a two-sample Kolmogorov–Smirnov test indicated that their slopes’ distributions were significantly different (*p* < 0.01). Both histograms show a prevalence of 1 dB/dB slopes for low stimulus levels (< 70 dB SPL) and much steeper slopes (> 2 dB/dB) for high-stimulus levels (> 80 dB SPL). However, a slow DPOAE growth (slopes < 1 dB/dB) was found exclusively in the histograms corresponding to the low-frequency region, which is consistent with the compressive nonlinearity typical of DPOAE growth functions from the frog’s AP (Meenderink and Van Dijk 2004; Vassilakis et al. 2004). In contrast, linear growth at a rate > 1 dB/dB prevails in the high-frequency region consistent with the DPOAE growth functions from the BP (Meenderink and Van Dijk 2005, 2006).

In males, slope analysis included the three sensitivity peaks that appeared in the low-, mid- and high-frequency regions of the colormaps. In general, results were similar to those found in females. Slope distributions from the low and mid-frequency regions, presumably corresponding to the AP, are skewed toward shallow slopes (≤ 1 dB/dB), while the slope distribution corresponding to the high-frequency region, presumably corresponding to the BP, appeared skewed toward steeper slopes (~ 3 dB/dB) which is consistent with a passive DPOAE generation (Meenderink and Van Dijk 2006).

### Effects of ET closure on inner ear tuning

**Fig. 6:**
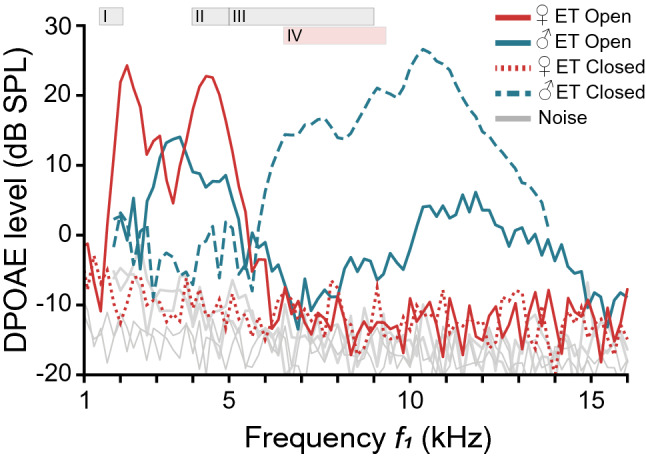
Changes in inner ear sensitivity with the ET closure. DP-grams registered in a female (red) and a male (green) with the ET open and closed. In females, the DPOAE response disappeared after closing the ET. In males, the ET closure caused a sensitivity shift towards the high frequencies; DPOAEs attenuated at low frequencies and increased at high frequencies relative to the amplitudes recorded with the ET open. Shaded rectangles (top) represent the ranges of the fundamental frequency of *Odorrana tormota* vocalizations (male: gray, female: pink): I meow and staccato calls, II shallow-fm calls, III single and multi-note calls, IV female calls (data from Feng et al. 2009; Zhang et al. 2017)

For a small subset of frogs (2 females, 4 males), we investigated the effect of ET closure on inner ear sensitivity. DP-grams were recorded (as described above) with the ET open and closed. No DPOAEs were detected in females with the ET closed (Fig. 6a). By reopening the ET, the response recovered completely indicating that the ET closure procedure did not affect hearing permanently. The disappearance of the DPOAE response is understandable if we analyze the electrophysiological, vibrometric and DPOAE data available for females of this species. Shen et al. (2011) evaluated single-unit responses from the torus semicircularis of *Odorrana* females and only found units with best frequencies < 10 kHz. DP-grams recorded with the ET open showed that the spectral sensitivity of the inner ear of these female frogs is limited to frequencies below 10 kHz. By closing the ET, the middle ear tuning changes to a frequency range (> 10 kHz) where the inner ear and even the central auditory system have limited or no sensitivity (this study, Shen et al. 2011). Taken together these results suggest that ET closure temporarily impairs hearing in *Odorrana* females.

In contrast, DPOAEs in males do not disappear in the noise with ET closure but undergo a shift in inner ear sensitivity (Fig. 6b). DP-grams recorded with the ET closed showed amplitude changes in the relative maxima: DPOAEs attenuated (median − 14 dB) at low frequencies and increased (median + 8 dB) at high frequencies relative to the amplitudes recorded with the ET open. This shift in the DP-grams towards the high frequencies suggests that the ET closure not only changes the middle ear tuning and inward sound transmission through the ossicles, but also affects outward transmission of the otoacoustic emissions generated by the inner ear amplification. Considering that maximum DPOAE amplitudes generally correlate to the frequencies of high auditory sensitivity (Kössl 1992; Van Dijk and Manley 2001; Bergevin et al. 2008), our results indicate that the ET closure also impacts hearing in *Odorrana* males but enhances sound perception at the higher frequencies.

The present study revealed the inner ear sensitivity of both sexes of *O. tormota*, an anuran species in which both sexes vocalize. Sexual differences in tuning, previously described at other levels of the auditory system (Shen et al. 2011), are now shown in the inner ear indicating that each sex is more sensitive to the frequencies of the other sex's vocalizations. Moreover, we confirmed that the capability of closing the ET is not only present in males but also in females of *O. tormota*. The ET closure seems to play a functional role in hearing; it impacts the sensitivity of middle and inner ear at frequencies used for social and sexual communication with conspecifics. In males, an increased sensitivity at higher frequencies would presumably confer advantages in locating calling females in the acoustically complex environment where calls of conspecific males compete with high levels of low-frequency noise (i.e., the sound of flowing water). We speculate that in females, the ET closure might play a role in hearing protection since it causes the disappearance of the inner ear response, but further investigation to verify this is required. This study broadens our understanding of anuran hearing and the diversity of adaptations to acoustically communicate in noisy environments.
